# DC electrical stimulation enhances proliferation and differentiation on N2a and MC3T3 cell lines

**DOI:** 10.1186/s13036-022-00306-8

**Published:** 2022-10-13

**Authors:** Daniel Martín, J. Bocio-Nuñez, Santiago F. Scagliusi, Pablo Pérez, Gloria Huertas, Alberto Yúfera, Mercè Giner, Paula Daza

**Affiliations:** 1 Electronics Technology Department, Universidad de Sevilla, Seville, Spain; 2 Microelectronics Institute of Seville, Universidad de Sevilla, Seville, Spain; 3 Bone Metabolism Unit, UGC Medicina Interna, HUV Macarena, Seville, Spain; 4 Electronics and Electromagnetism Department, Universidad de Sevilla, Seville, Spain; 5 Departamento de Citologia e Histologia Normal y Patologica, Universidad de Sevilla, Seville, Spain; 6 Cell Biology Department, Universidad de Sevilla, Seville, Spain

**Keywords:** Cell differentiation, Electrical stimulation, Microelectrodes, Tissue engineering

## Abstract

**Background:**

Electrical stimulation is a novel tool to promote the differentiation and proliferation of precursor cells. In this work we have studied the effects of direct current (DC) electrical stimulation on neuroblastoma (N2a) and osteoblast (MC3T3) cell lines as a model for nervous and bone tissue regeneration, respectively. We have developed the electronics and encapsulation of a proposed stimulation system and designed a setup and protocol to stimulate cell cultures.

**Methods:**

Cell cultures were subjected to several assays to assess the effects of electrical stimulation on them. N2a cells were analyzed using microscope images and an inmunofluorescence assay, differentiated cells were counted and neurites were measured. MC3T3 cells were subjected to an AlamarBlue assay for viability, ALP activity was measured, and a real time PCR was carried out.

**Results:**

Our results show that electrically stimulated cells had more tendency to differentiate in both cell lines when compared to non-stimulated cultures, paired with a promotion of neurite growth and polarization in N2a cells and an increase in proliferation in MC3T3 cell line.

**Conclusions:**

These results prove the effectiveness of electrical stimulation as a tool for tissue engineering and regenerative medicine, both for neural and bone injuries. Bone progenitor cells submitted to electrical stimulation have a higher tendency to differentiate and proliferate, filling the gaps present in injuries. On the other hand, neuronal progenitor cells differentiate, and their neurites can be polarized to follow the electric field applied.

## Background

Functional tissue generation through tissue engineering, by applying electrical stimulation (ES), has a high impact in various areas of regenerative medicine [1]: the restoration of damage caused in the nervous system [2], systemic muscle disease and localized muscle damage [3], or the correction of bone defects (with or without prosthetic elements) by producing functional osteoblasts [4, 5]. Cell differentiation and tissue engineering represent a great challenge for the future, facing the creation of biological material from cell cultures, which will allow the development of therapies to alleviate various diseases.

In the context of obtaining differentiated nerve cells, neuroblastoma is a type of cancer that develops in primitive nerve cells, neuroblasts, which remain in the body as residual of the embryonic phase of development; it generally affects children and it is formed in nervous tissue, but it generally starts in the adrenal glands [6, 7]. The neuroblastoma cell line 2A or N2a (also N2A or neuro-2a) is derived from albino mouse neuroblastoma cells and comes from a spontaneous tumor of a strain of *Mus musculus*. This cell line is commonly used for research on neuronal signaling pathways, axon growth, and trophic interactions between neurons, any fundamental aspect related to the development of the nervous system as well as for the treatment of nerve injuries. This cell line has the advantage of changing its morphology by differentiating and has been very useful to test certain neurotoxic drugs and compounds with chemotherapeutic possibilities [8–10]. On the other hand, it is remarkable the increase of situations related to bone pathology that demand a clinical solution. These include, among others, important bone tissue defects, traumatic fractures with lack of consolidation, tumor resections, debridement of septic or aseptic necrotic tissues; also, the increase in prosthetic replacements, due to degenerative or metabolic bone disease, which is increasingly widespread in elderly people, with less ability to regenerate bone tissue and ensure future biomechanics; and finally, the wide use of dental bone implants occurring at increasingly precocious ages, that require a long half-life and lead to an improvement of quality of life. Bone tissue engineering is gaining great interest as an alternative approach to treatments aimed at bone regeneration, as consequence of the proliferation, maturation, and differentiation of osteoblast cells (OB) [11]. Factors influencing the stimulation of these processes, chemical or physical, are of great interest for bone tissue regeneration and prosthesis development. The aim of this work is to study the behavior of neuroblastoma and osteoblast cells after applying electrical stimulation using N2a and MC3T3 cell lines.

Electrical Stimulation (ES) applied to neural tissues tries to mimic the endogenous electric fields present in them, with amplitudes in the range of millivolts per millimeter, producing effects such as: proliferation [12, 13], neurite outgrowth [2, 14], differentiation of neural stem cells [2, 12, 15], and others. The combination of stem cells and electrical stimulation has been proved to be a valid approach for nerve regeneration in vivo [16, 17]. The effects of ES on proliferation and differentiation are also very suitable for improving osseointegration and osteogenesis in bone implants [18] as well as injuries [19]. Osteogenesis has been previously induced in cell cultures: using conductive scaffolds [20–22] or capacitive coupling with electrodes introduced from the top [23–28]. Both animal and plant cells can react to electric fields (EF) growing or migrating in certain directions. In [29], it was illustrated that for electric fields applied in the range of [0.1, 1] mV/cell diameter it is possible to find some type of response in a wide range of cells studied. Lower threshold sensitivities to alignment with the applied EF were observed in N2a at [0.35, 50] mV/mm, as well as the proliferation of neurites in the presence of a constant EF (DC). A similar behavior was observed in myoblasts [30], where these were aligned perpendicular to the applied EF. In [2], a normal electric field was applied to the surface of a N2a culture, the maximum length of differentiated neurites (90 μm) was obtained at the optimal electric field amplitude of 250 mV/mm. Studies with N2a also characterize the response to various doses of dopamine, using electrical impedance spectroscopy (EIS) [31]. For osteoblasts, we have also found works that prove the functional improvement of long-term behavior when subjected to biphasic electrical pulses [32], using commercial electrical stimulators on the surfaces of titanium nanotubes. Other authors [4] apply constant EF, achieving optimal effects for certain values of field amplitude (200 mV/mm), avoiding oxidation-reduction reactions at the electrodes surface [2], that can generate cytotoxicity. ES is also applied to other cell lines, such as cardiac precursor cells, because of its effects on differentiation and applications on tissue engineering [33–36].

Many proposals study neuronal or muscular electrical stimulation in order to activate the defective nervous system or regenerate its activity [37], but this is not our case. In the context of cell differentiation, the use of direct current (DC) as well as alternating current (AC) circuits is described. Although electrodes generally are in direct contact with the culture medium, it is also possible to apply EFs by means of two electrically isolated capacitive plates from the culture medium or tissue (Capacitively Coupling Electric Field, CCEF) [2]. N2a assays apply constant EF (DC) of different magnitudes: in the range [0.1, 1.0] mV/cell diameter [29]. On the other hand, for myoblast and osteoblast cell stimulation, AC/DC signals are used, with various characteristics [3, 4, 38], estimating that at the cellular level, values of tens or hundreds of mV or μA (difference of potential between two points of the cell or currents supported by them) are suitable for cell stimulation [38], and sufficient to activate EF sensitive proteins (channels and receptors of the cell membrane), modify gene expression, alter cellular communication, etc. In these studies, the effect caused by ES is not evaluated until it is stopped, the culture is removed from the incubator or bioreactor, and the cultures are analyzed [2]. In these conditions, it is not possible to establish in real time whether the ES signal is working optimally, or if it is necessary to alter the parameters (amplitude, frequency, duty cycle, etc.) to better activate the cellular response. Moreover, it is not possible to be sure that the culture has not been damaged by an inappropriate signal (excessive level of voltage, intensity, or energy).

This work presents the main experimental results obtained in ES assays with DC electric fields applied to N2a and MC3T3 cell lines. The final purpose of the ES electronic circuit is to allow an easy control of the voltage applied to the cell lines, offering the possibility, when necessary, of tuning the ES parameters depending on the progress of the cell culture. In previous works, bare electrodes were introduced at the cell culture medium, adding uncertainty about the real electric field that it was being applied to each individual electro-stimulated cell. Pursuing this challenge, we present, in an initial stage, a first estimation of the influence of DC EF on the two cell lines selected. The setup employed has been built using commercial interdigitated electrodes (IDEs) (Applied Biophysics; Troy, USA). Unlike previous approaches reported, these IDEs were selected because the position and configuration of their electrodes ensures that electrically stimulated cells receive the EF generated fully.

## Methods

### Experimental setup

The in vitro experimental setup included several blocks: the signal generation circuit, the cell culture plate with electrodes, and the 3D printed bed that holds the connections to the circuit and the culture chambers.

**Fig. 1:**
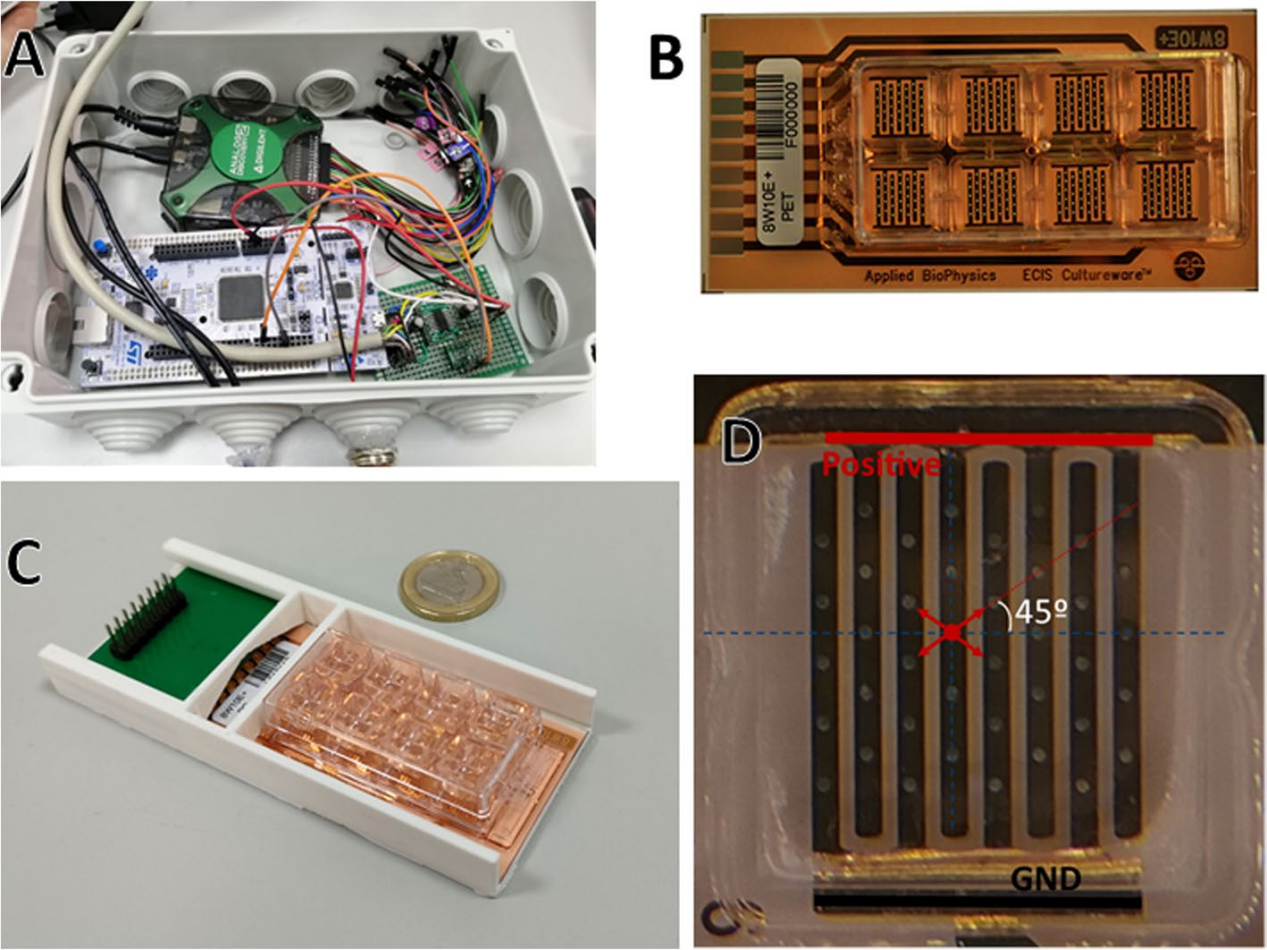
Experimental setup. **A** Circuitry inside the enclosure. **B** 8W10E+ cell culture plate. **C** Cell plate and connector PCB in the 3D printed bed. **D** Close up of a well in an 8W10E+ plate with annotations highlighting the direction of the strongest field lines and the electrodes configuration in space

The circuit designed consisted of a USB oscilloscope working as a DC power supply (Analog Discovery 2, Digilent, Washington, USA), that allowed us to obtain a low noise DC signal, and an amplification stage. The amplification stage was powered at + − 5 V and employed OPA4228 operational amplifiers (Texas Instruments, Texas, USA) at inverter amplifier configuration to generate the desired DC signals from the negative supply voltage. The inverter amplifier gains were set by resistance ratios that ultimately define the DC voltage levels outputted to the cell culture. The amplification stage was soldered on a perforated board, all circuitry was encapsulated in a hermetic enclosure to protect it from the environmental conditions of the cell incubator (Fig. 1A).

In this study, 8W10E+ cell culture plates from Applied Biophysics (Troy, USA) were used (Fig. 1B). These plates are made of a clear polycarbonate substrate and have 8 wells, each with two sets of 20 round interdigitated transparent gold electrodes: one set positive and the other set to ground. The electrodes have a diameter of 250 μm and a separation of approximately 1 mm between the closest ones; this means that all applied voltages can be directly expressed in mV/mm. These plates allow us to be completely sure about the EF suffered by the cells, since there are 20 positive (and 20 ground) EF application points on the culture substrate and cells rest on top of them, electrically stimulated cells will sense the same or similar EF. The configuration of the electrodes in space imposes the weakest field lines at 90°, while the strongest ones are located around 45° and 135°, in reference to the electrode (Fig. 1D).

The 3D printed bed was designed to hold both the cell culture plate and a printed circuit board (PCB) that connects it with the signal generation circuit. The PCB has 9 connectors that make contact with the gold fingers on the 8W10E+ plate and a pin header connector to interface with the signal generation circuit (Fig. 1C).

### Cell and culture conditions

#### N2a

N2a cells were generously provided by Dr. Diego Ruano (Institute of Biomedicine of Sevilla, Sevilla, Spain). Cells were cultured in medium consisting of 50% DMEM High glucose (Biowest, Nuaillé, France) and 50% Opti-MEM (Gibco, Alcobendas, Spain) supplemented with 10% (v/v) fetal bovine serum (FBS) (Gibco, Alcobendas, Spain), 2 mM L-glutamine, 50 μg/mL streptomycin, and 50 U/mL penicillin (Sigma-Aldrich, Madrid, Spain). 25.000 N2a cells were seeded on 500 μL of completed medium in each of the 8 wells of an 8W10E+ plate; this number of cells ensures the optimal density: plenty of cells to form a layer and with enough space for cells to differentiate properly. The day before electrical stimulation, serum-free medium was added, replacing the completed medium. Cells were maintained at 37 °C in a humidified atmosphere with 5% CO2 and they were routinely subcultured in order to be in exponential growth phase when used for experiments. Each experiment was independently performed at least in triplicate.

#### MC3T3

MC3T3E1, a murine pre-osteoblast cell line (CRL-2593, from ATCC, Manassas, VA, USA) was used to evaluate the effects of ES on proliferation, viability, and differentiation process. Cells were cultured in Minimum Essential Medium (MEM), containing 10% fetal bovine serum plus antibiotics (100 U/mL penicillin and 100 mg/mL streptomycin) (Invitrogen) and antifungal (amphotericin B 100 mg/mL (BioWittaker Lonza). Osteoblasts cells were seeded at a cellular density of 175,000 cells per condition. Plates were kept at 37 °C in a humidified 5% CO2 atmosphere.

At 72 h of osteoblast culture, the cell medium was changed to osteogenic media (α-MEM medium) supplemented with 10 mM ascorbic acid (Merck, Germany), 10 mM of β-glycerophosphate (StemCell Technologies, Canada) and 10 nM of dexamethasone (Sigma). In-vitro experiments were carried out at 4, 7 and 10 days of cell incubation, in which the supernatants were transferred to vials to be stored at − 80 °C until the last day of the experiment, to evaluate cell differentiation.

### Electrical stimulation protocol

N2a cells were stimulated for 6 hours and left in serum-free medium for another 18 hours. MC3T3 cells were stimulated 3 hours every day for 11 days; the medium was refreshed every other day before stimulation. The stimulation signals consisted of DC voltages of amplitudes: 0 (control), 125, 250 and 500 mV/mm for both cell lines; conditions were duplicated in 2 wells of the culture plate, thus using the 8 wells of the 8W10E+ plate. For MC3T3 cells, two identical plates were seeded and stimulated, a higher cell number was required in order to perform the genetic study. This stimulation protocol was designed based on previous works (Table 1). Works aimed at neural differentiation and neurite outgrowth apply DC EF for 1 to 8 hours, most often in a single day, with amplitudes lower than 1 V/mm. For osteogenesis, the DC amplitudes used are similar, but the number of days of ES is larger, ranging from 3 to 14 days.

**Table 1:** Several references of Electrical Stimulation conditions reported using DC signals, on neuroblastoma and osteoblast cell lines

Cell Line	Amplitudes (mV/mm)	ES duration		Effect	Ref
		Hours/day	Days		
N2a	110, 250, 500, 750, 1000	6	1	Differentiation and neurite outgrowth	[2]
Dorsal Root Ganglia Neurons	50	8	1	Neurite outgrowth	[39]
PC12	3000	1	1	Neurite polarization and outgrowth	[40]
Neural precursor cells (NPC)	115	2	2	Migration	[41]
Immortalized keratinocytes HaCaT	150	1	1	Proliferation and migration	[42]
MC3T3	100	1, 2	3	Differentiation and Osteogenesis	[26]
Rat bone marrow stromal cells	3.5, 35, 350	2, 4, 12	14	Osteogenesis	[22]
hMSC	100	0.167 (10 min)	21	Differentiation and osteogenesis	[23]
hMSC	100, 200	1	21	Proliferation, differentiation and osteogenesis	[24]
Rat mesenchymal stem cells	100	1	14	Osteogenesis	[28]

### N2a counting method and differentiation analysis

**Fig. 2:**
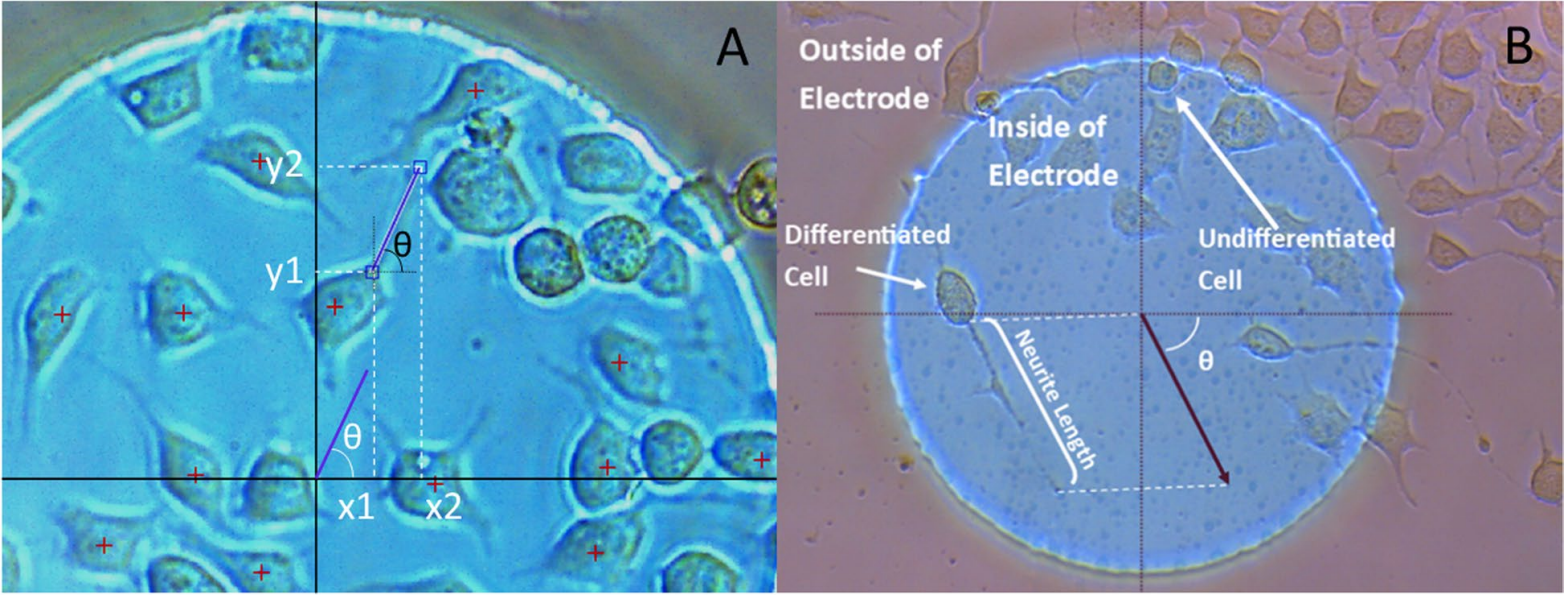
**A** Snapshot of the Matlab program developed to count cells and measure neurite length with annotations that illustrate the process of calculating the orientation of neurites. Red crosses indicate points where differentiated cells have been counted. **B** Example of one of the images taken with annotations that illustrate some of the guidelines followed in the counting process

After the stimulation protocol was performed, images of each electrode were taken in an inverted microscope (Leica, Wetzlar, Germany) with a 20X objective. The images were then analyzed: undifferentiated and differentiated cells were counted, neurites were measured, and their orientation was also assessed. In order to measure neurite orientation, images were stabilized taking the electrode as reference, and angles were extracted from the coordinates of the neurites ends. For this purpose, eq. (1) was used, where θ represents the orientation of the neurite and (x_1_, y_1_) and (x_2_, y_2_) are the coordinates of the ends of the neurite (Fig. 2A).

1 $$\theta ={\mathit{\tan}}^{-1}\frac{y_2-{y}_1}{x_2-{x}_1}$$

Only cells on top of the electrode surface were considered (Fig. 2B); in this way we ensure that the counted cells had fully experienced the electrical stimulation delivered. A MATLAB program was developed in order to analyze these images [43], the cited program allows one to click on cells to count them and displays a ruler tool with moving ends to measure the length of neurites (Fig. 2A). In the whole process, four steps were necessary to obtain the results: a) firstly, all cells were counted; b) secondly, only differentiated cells were selected; c) thirdly, the images were stabilized in order to do the last pass d) fourthly, neurite length and orientation were measured with the ruler tool.

### N2a Inmunofluorescence assay

An additional experiment was carried out for the immunofluorescence assay on N2a cells. Cells were stimulated for 6 hours at 250 mV/mm, they were then incubated for 18 h in serum-free medium, after which they were fixed with 4% paraformaldehyde (Fluka, Honeywell International) in PBS at room temperature for 15 minutes and permeabilized with 0.5% Triton X-100 (Merck KGaA, Darmstadt, Germany) in PBS at room temperature for 5 minutes. Samples were blocked in PBS with 0.1% Tween20 (Sigma-Aldrich, Madrid, Spain) and 1% bovine serum albumin (Sigma-Aldrich, Madrid, Spain). A primary monoclonal Anti-α-Tubulin (Sigma-Aldrich, Madrid, Spain) was diluted 1:3000 in the solution used for blocking, cells were kept in this solution for 45 minutes at room temperature. Goat Polyclonal secondary antibody to mouse IgG (H&L) - Alexa Fluor 488 was diluted 1:500 in the same blocking solution and incubated for 30 minutes at room temperature. Epifluorescence microscopy was performed using an inverted Leica microscope with a 10X objective (Leica, Wetzlar, Germany).

### MC3T3 cell viability and proliferation assay

In order to quantify the proliferation of MC3T3 an AlamarBlue assay was carried out the day after the last stimulation protocol (day 10). Briefly, 300 μL AlamarBlue mixture containing 10%AlamarBlue™ stock solution (ThermoFisherScientific, UK) and 90% cell culture medium was added directly to the samples in a culture plate with electrodes, then incubated at 37 °C for 120 min. Following incubation, three 50 μL replicates of the aliquots were transferred from each sample into a clear 96 well plate. A sample control (media plus AlamarBlue™ reagent) was required on this assay. Fluorescence readings were taken using a TECAN, Infinity 200 Pro microplate reader at 570 nm excitation.

### MC3T3 differentiation by alkaline phosphatase (ALP)

MC3T3 differentiation levels were evaluated through alkaline phosphatase (ALP) activity, using the Alkaline Phosphatase Assay Kit Colorimetric (Abcam ab83369, Abcam, Cambridge, UK). MC3T3 medium was refreshed every other day before electrical stimulation. The used medium was stored at − 80 °C until the last day of stimulation. The assay was performed at 3, 4, 5, 6, 7, 10 and 14 days, by triplicate, according to manufacturer’s protocol. The absorbance at 405 nm of 4-nitrophenol was measured in a 96-well microplate reader. Data were expressed as U/mL of pNPP (para-Nitrophenylphosphate).

### Real-time PCR analysis on MC3T3

RNA was isolated from MC3T3E1 cells by using Trizol, following manufacturer’s instructions (Invitrogen, CA, USA). To carry out the Real-time PCR, total RNA was reverse-transcribed with RT First strand kit (SABioscience, USA) according to manufacturer’s instructions.

To validate the PCR assay, Real-time PCR was done with cDNA from each condition, and was repeated three times for each gene using SYBRGreen (Roche, Switzerland) and the primers of *Runx2*, osteoprotegerin (*OPG)*, *Ostx* and *18S rRNA* (housekeeping gene) were provided by QuantiTectPrimer Assay (Qiagen, USA). Real-time PCR were performed in Step-One system (Applied Biosystems, USA).

### Statistical analysis

All statistical analyses were performed using one-way analysis of variance (ANOVA) test, where *p* values < 0.05 were considered statistically significant. All tests have been performed with reference to the control condition.

## Results

### Density of N2a cells

**Fig. 3:**
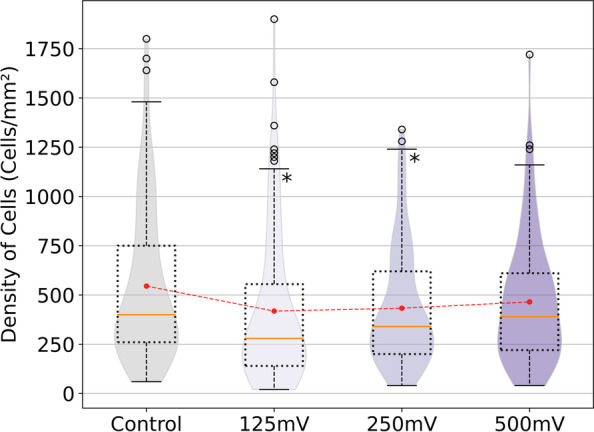
Density of N2a cells, calculated as the number of N2a cells per square millimeter. Data is displayed as a boxplot and a violin plot overlapped. Orange lines represent median values, and black dots represent outliers. Mean values are displayed as red dots connected by a dashed line. (*, *p* < 0.05; *n* = 435)

As an approach to cell viability, we obtained the density of N2a cells expressed as the number of N2a cells per square millimeter, this result is shown in Fig. 3. To obtain this result the number of cells counted on each image has to be divided by the area of the electrode, since this is the area that is considered when counting. N2a cells were stimulated for 6 hours at 4 different DC voltages (0, 125, 250 and 500 mV/mm). As it can be observed, a significant difference was found between 125 and 250 mV/mm respect to the control; the number of cells per square millimeter decreased from an average of around 600 cells/mm^2^ in the control to approximately 450 cells/mm^2^ in both conditions. The other stimulated condition (500 mV/mm) also shows a decrease with respect to the control, although it was not as pronounced as the other two ES conditions. Overall, all ES conditions have a smaller number of cells per surface than the control.

### N2a morphology and distribution

**Fig. 4:**
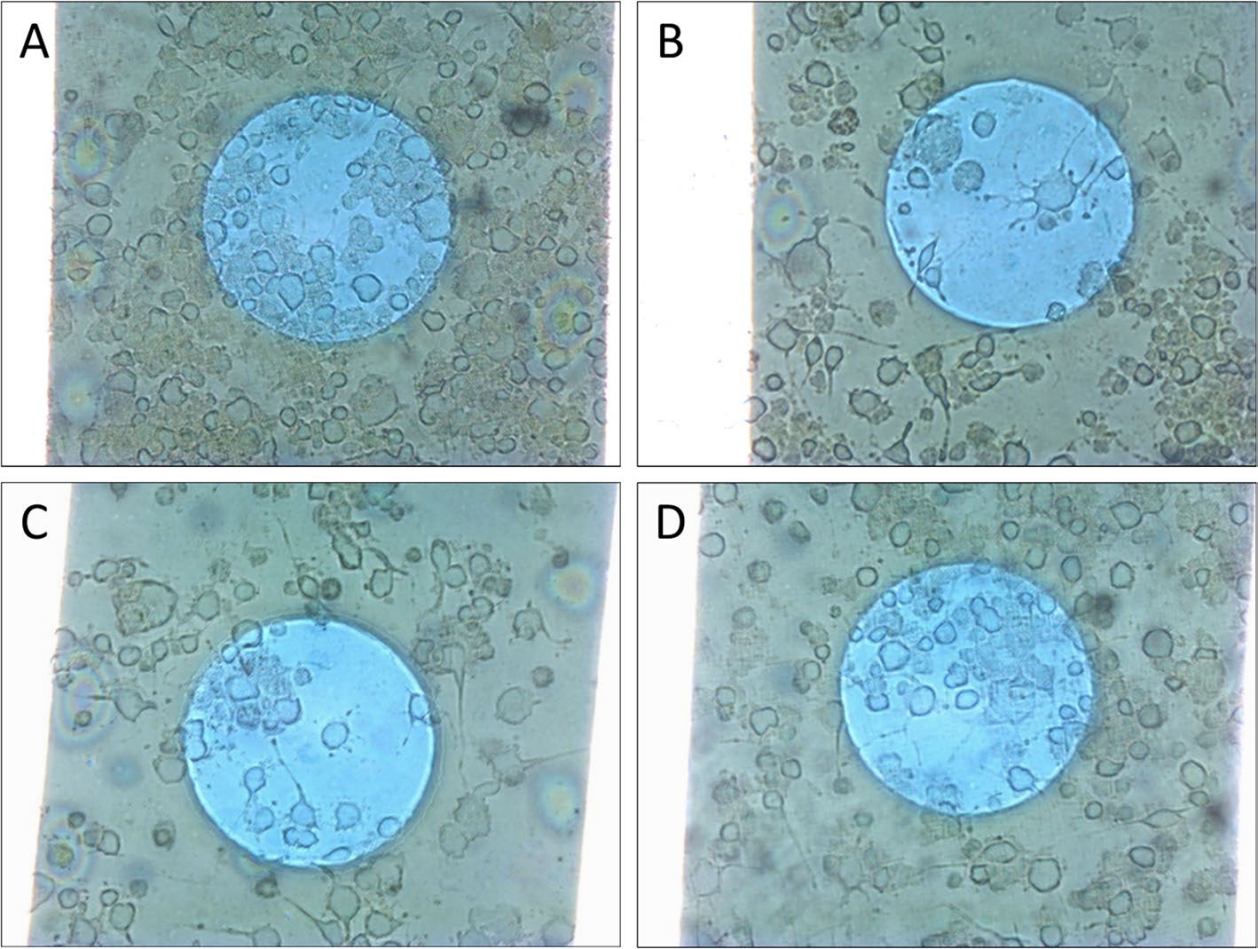
Representative images of N2a cells taken with a 10X objective after 6 hours of electrical stimulation. **A** Control. **B** 125 mV/mm. **C** 250 mV/mm. **D** 500 mV/mm

N2a cells change morphology while they are differentiating; they develop extensions called neurites and become more elongated. Once N2a cells differentiate they do not divide any more. Figure 4 illustrates four of the images used in the counting and differentiation analysis, one for each condition ordered by amplitude (Control, 125, 250 and 500 mV/mm). In the first image, representing the control condition, most cells are round, and density is the highest of all (Fig. 4A). Secondly, 125 and 250 mV/mm conditions are represented; cell morphology and density are very similar, with cells showing neurites and a lower density (Fig. 4B and C). The last image represents cells after 500 mV/mm treatment and it presents a middle ground between the control and the other two stimulated conditions, with some differentiated cells and a slightly higher density than in the other two ES conditions (Fig. 4D).

**Fig. 5:**
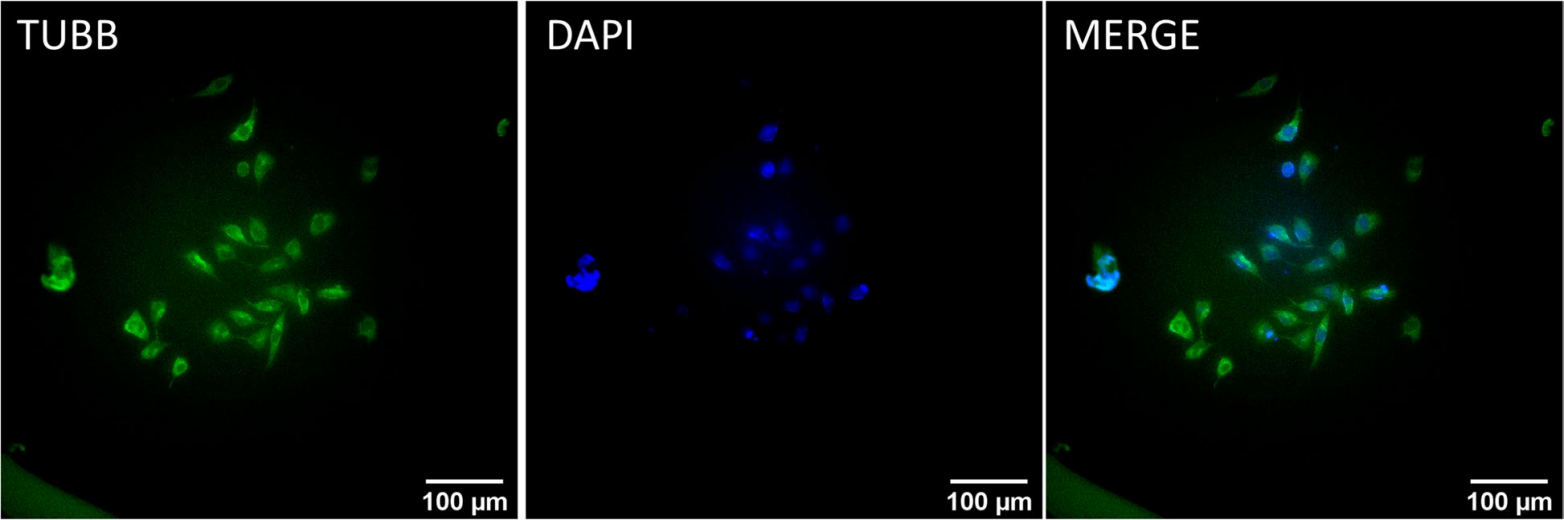
Immunofluorescence images of N2a cells stimulated for 6 hours at 250 mV/mm

To further asses N2a morphology, an immunofluorescence assay was carried out, using α-Tubulin /DAPI staining. The immunofluorescence assay was aimed at tubulin, a cytoskeleton essential protein that plays a vital role in the development of neurites. The images displayed in Fig. 5 correspond to N2a cells stimulated at 250 mV/mm for 6 hours; cells appear to have a differentiated morphology, mostly elongated with neurites.

### N2a differentiation

**Fig. 6:**
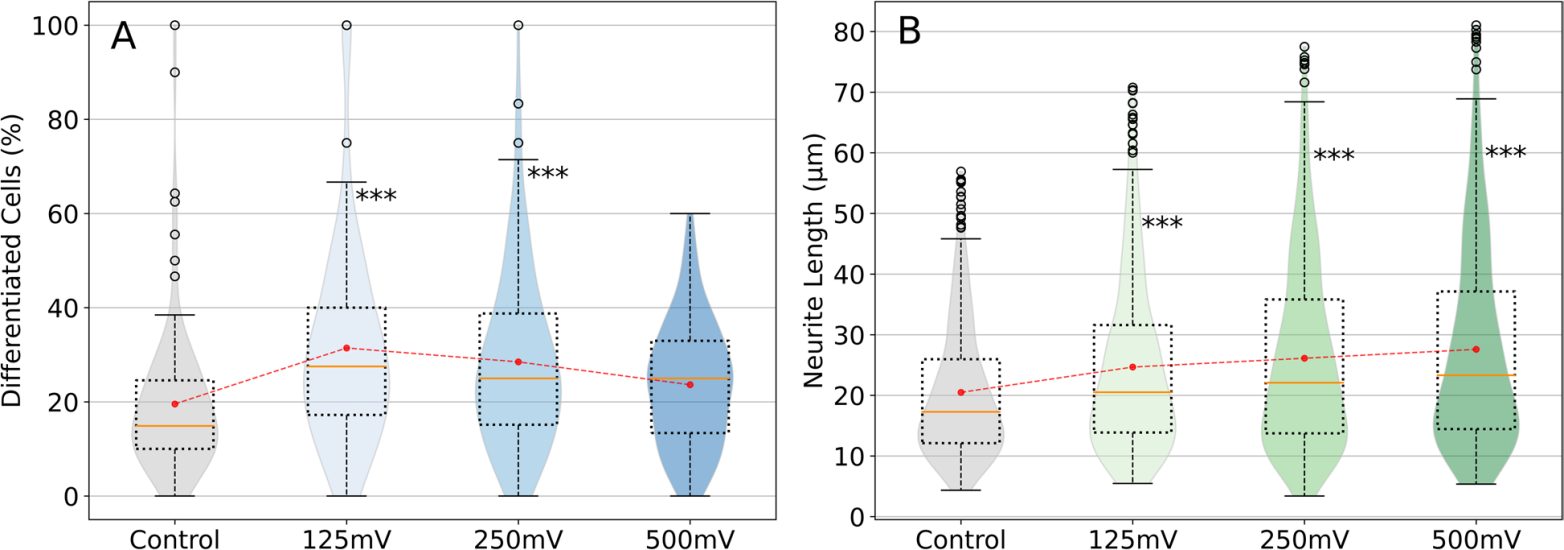
Results of the differentiation analysis of N2a cells. **A** Percentage of differentiated N2a cells, calculated as the number of differentiated cells divided by the number of counted cells multiplied by 100. Data is displayed as a boxplot and a violin plot overlapped. Orange lines represent median values, and black dots represent outliers. Mean values are displayed as red dots connected by a dashed line. (***, *p* < 0.001; *n* = 435). **B** Neurite length (μm) of N2a cells. Orange lines represent median values, whilst red and black dots represent means and outliers, respectively. (*** *p* < 0.001; *n* = 2004)

Figure 6A shows the percentage of differentiated N2a cells. Orange lines represent median values, and black dots represent outliers. Mean values are displayed as red dots connected by a dashed line. Non-stimulated cells had a lower tendency to differentiate overall. 125 mV/mm (32%) and 250 mV/mm (28%) were found to be the only conditions that had a significant effect on this parameter with respect to the control. In 500 mV/mm (24%) the difference is not significant, but its mean and median values are higher than those of the control condition (19%). The peak sensibility appears to be between 125 and 250 mV/mm, higher values do not cause the same effect.

Neurite length was also measured; results are shown in Fig. 6B. Non-stimulated differentiated cells had neurites with an average length of about 22 μm. All stimulated conditions show a significant increase in length compared to the control, an average of 25 μm for 125 mV/mm, 26 μm for 250 mV/mm and 29 μm for 500 mV/mm. There is not much difference in neurite length between stimulated conditions; that said, it appears to increase slightly as the amplitude does, being 500 mV/mm the condition that most promoted neurite outgrowth.

**Fig. 7:**
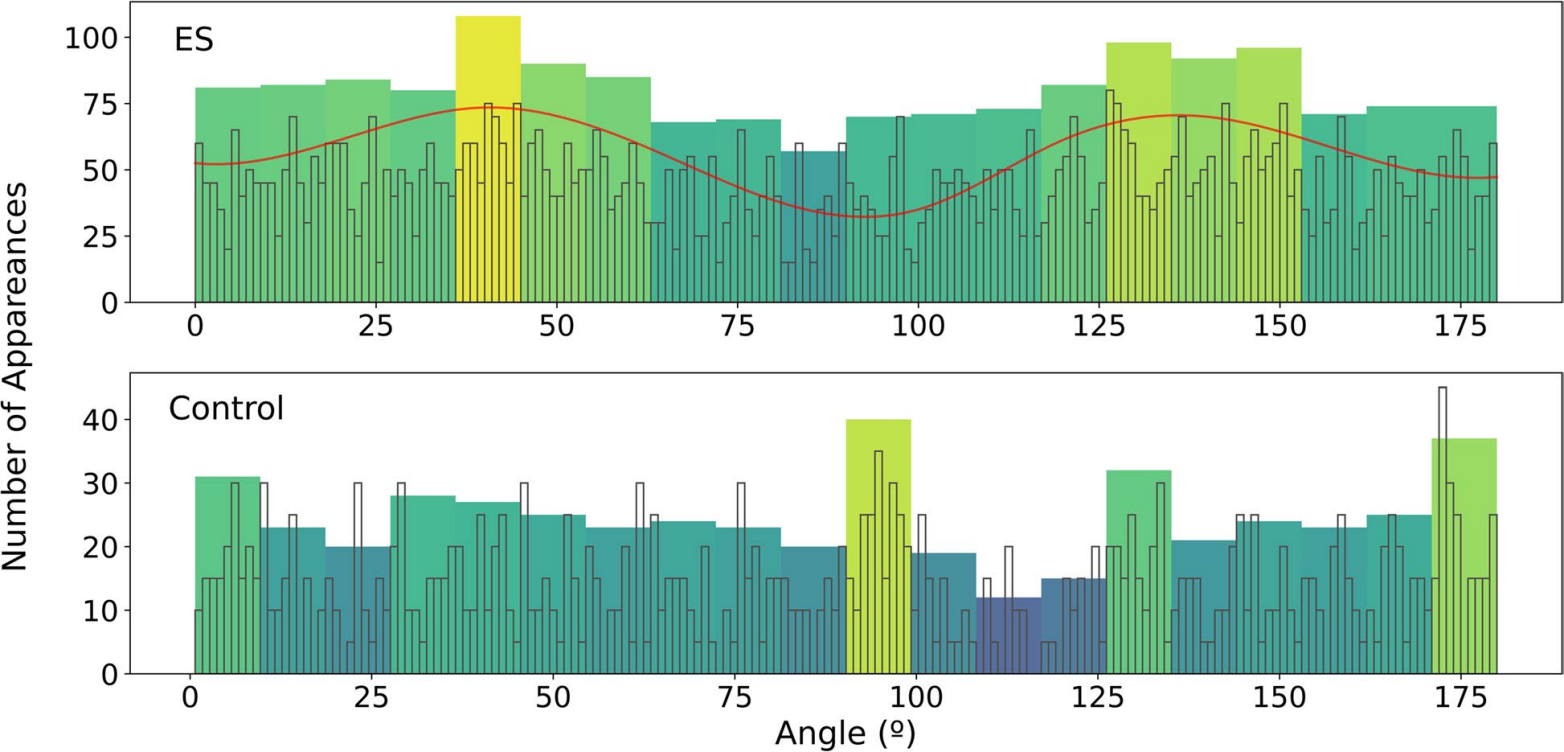
Neurite angles of stimulated cells (ES) and non-stimulated cells (control). Two histograms with different bin numbers are superposed in each axis, one with color and another without. The colored histogram has a bin number of 20 (groups of 9°) and acts as a low-pass filter for the distribution, whilst the uncolored histogram has 180 bins (every degree). Values of the uncolored histogram have been scaled by 5, to enhance visibility. A red hardcoded spline has been added to emphasize the shape of the distribution on the ES histogram

With the initial and final points of the neurite, the angle can be calculated using the inverse of the tangent, these results are shown in Fig. 7 as a histogram. All electrical stimulated conditions have been merged on the top axis (ES) whilst the control is shown on the bottom axis. The distribution of stimulated cells shows two hills: one around 45° and another near 145°, and a valley in the surroundings of 90°. The two hills coincide with the direction of the strongest field and 90° represents the direction in which ES is weakest. It appears that cell neurites have responded to the EF polarizing in the direction where the field is strongest. The distribution of angles in non-stimulated cells is more chaotic, it has a slightly decreasing trend until it reaches a minimum around 115°.

### MC3T3 AlamarBlue assay

**Fig. 8:**
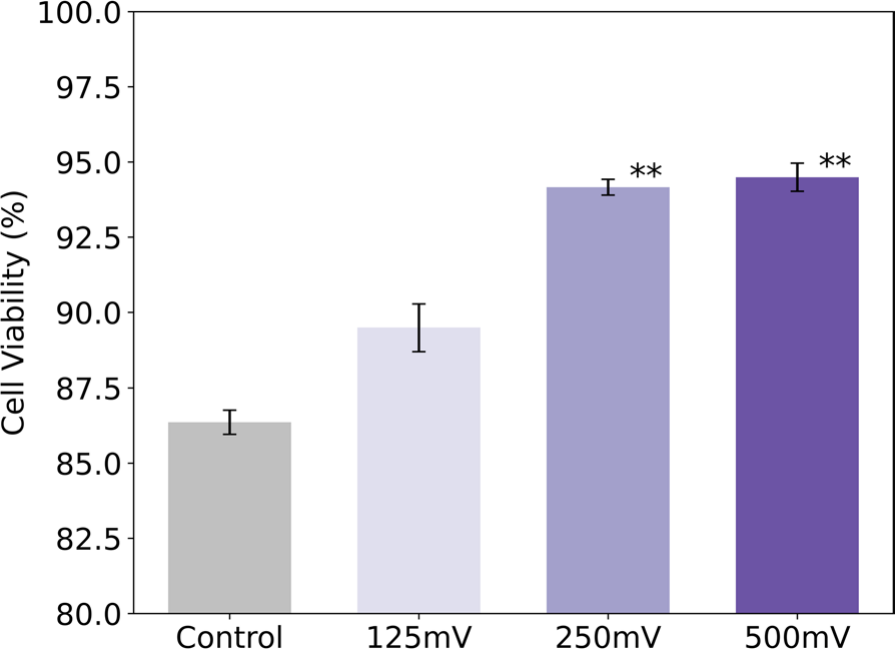
Results of the viability analysis of MC3T3 cells expressed as percentage, it was calculated as the absorbance of the reduced and oxidized product of AlamarBlue. (**, *p* < 0.01; *n* = 2)

To examine the cell viability in MC3T3 cultures, we performed analyses with AlamarBlue, after 11 days of electrical stimulation (Fig. 8). We observed that there was an increase in viability as the magnitude of DC electrical stimulation increased, being this more present at 250 mV/mm (94.2%) and 500 mV/mm (94.5%) vs control (86.3%) and 125 mV/mm (89.5%). It appears that viability increases with the amplitude until it stabilizes at the 250 mV/mm condition.

### MC3T3 ALP activity

**Fig. 9:**
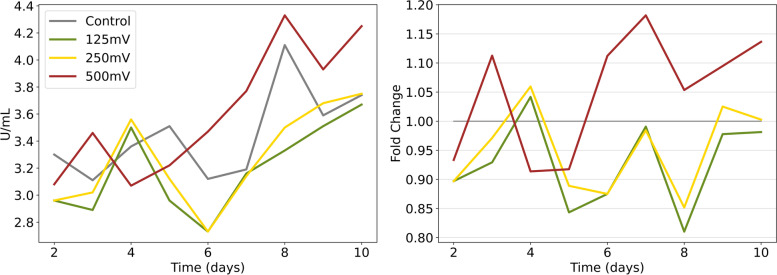
ALP activity (left) and ALP activity normalized to control (right); measured in days 3 to 14 of ES. Conditions are shown as lines of different colors

ALP is an early marker of osteogenic differentiation. ALP activity is represented in U/mL and as fold change with respect to the control in Fig. 9. The first measure (day 1) has not been considered since the measurement was taken before ES. All conditions show an increasing tendency on the second half of the experiment, from day 6 on. 125 and 250 mV/mm have a similar behavior overall and have a lower value than the control on most days; 500 mV/mm, on the other hand, starts to stand out at day 6 and keeps being the top condition from then on.

### MC3T3 real-time PCR

**Fig. 10:**
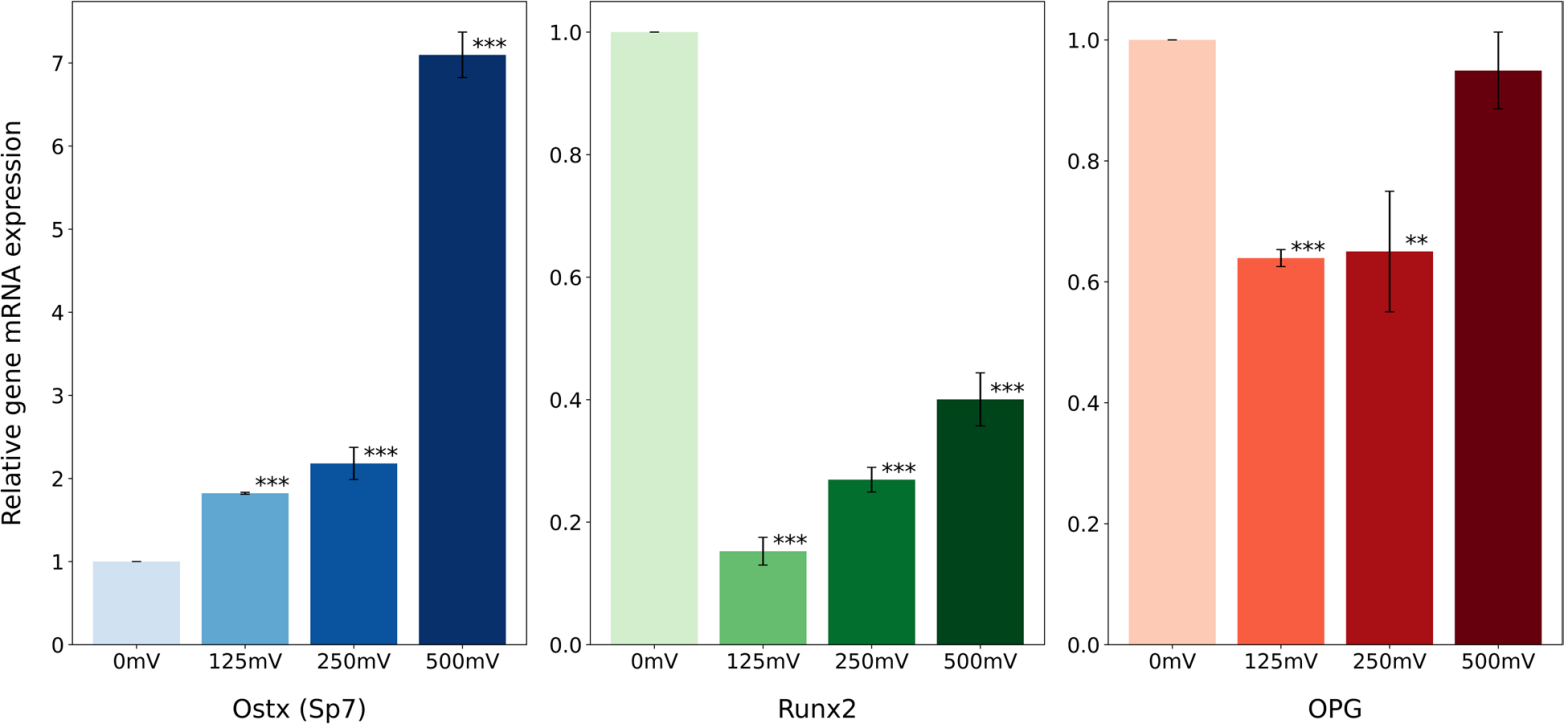
Quantitative real-time PCR analysis of the osteogenesis-related gene expression of OSX (a), RUNX2 (b), OPG (C) at 14 days of MC3T3-E1 cells cultured (**, *p* < 0.01; ***, *p* < 0.001; *n* = 3)

The expression of osteoblast markers genes OSTX, RUNX2 and OPG in MC3T3 cells subject to electrical stimulation for 11 days was evaluated in this study (Fig. 10). It is found that OSTX expression was upregulated for all ES conditions, following an increasing trend with amplitude, being maximum at 500 mV/mm. The other two genes showed a decrease in expression for ES conditions compared to the control, but followed the same growing trend associated with amplitude, this being more present in RUNX2.

## Discussion

This study aimed to evaluate the effects of DC electrical stimulation on N2a and MC3T3 cells, as a potential characterization for the design of DC electrical signals with optimal properties for cell differentiation. N2a cells were used to evaluate the impact of ES on neuronal differentiation, neurite outgrowth and alignment. On the other hand, MC3T3 cells were used as a model for osteogenesis and to determine cell proliferation, ALP activity and the mRNA level of osteosis-related genes. A stimulation system was designed and developed to deliver three different DC voltages to cells (125, 250 and 500 mV/mm), the stimulation protocol was chosen based on previous works and bibliography. The setup was designed considering commercial electrodes and off-the-shelf components.

### Differentiation of N2a cells is promoted by ES

ES based on DC voltages improved neuronal differentiation in N2a cells as hypothesized based on literature [2, 14, 15, 41], being 125 and 250 mV/mm the conditions with the highest percentage of differentiated cells. Cell density was also affected by ES, and stimulated conditions showed a decrease in this parameter with respect to the control; this was expected, since differentiated cells do not divide any longer and with those two conditions, we got more differentiation than the control. These results are in line with those obtained by [14], were the percentage of differentiated N2a cells was maximum at 100 mV/mm. Furthermore, in [15], the highest level of TUBB3 in a western blot, was also found at 100 mV/mm for another neuronal cell line, PC12.

Our results show that the length and orientation of the neurites were affected by the application of electric fields; neurites presented a tendency to grow in the orientation of the strongest field lines and grew larger than those of the non-stimulated condition. Neurite outgrowth due to electrical stimulation has been studied before [2, 14, 15, 39, 41], and our results are in agreement with these studies, but we would like to remark, that the effect of an electric field on neurite orientation has not been properly assessed. The setup used in our study allowed cells to be cultured on top of the electrodes that would deliver the ES. This electrode configuration forces cells to experience the full potential delivered, that is the reason why we chose it, because in this way, there is no uncertainty about the electric field experience by the culture. Furthermore, this electrode configuration also makes possible to know if the neurites were polarized by the EF, since the direction of the field is known.

In our opinion, the setup choice has been optimum since, as we have previously described, we have been able to ensure that electrically stimulated cells received the EF generated fully. In this way, we have established 125 and 250 mV/mm as the best conditions to differentiate neuroblastoma cells and that, neurites tend to grow in the orientation of the strongest field lines and grew larger than non-stimulated cells.

### Electrical stimulation enhances proliferation and differentiation of MC3T3 cells

The results show how, after 11 days of stimulation, there is an increase in osteoblastic proliferation dependent on the amplitude of ES. Other authors have also reported greater cell growth with amplitudes in that range, between 100 and 1000 mV/mm [44–47]. This correlates with a greater osteoblastic differentiation, since at 500 mV/mm we observed the highest ALP activity, in this condition, differentiation of the osteoblastic cell began after 6 days of stimulation.

RUNX2 and OSTX are two essential transcription factors for osteoblastic differentiation, RUNX2 has an earlier action, is first detected in pre-osteoblasts, and its expression is upregulated in immature osteoblasts, but downregulated in mature osteoblasts. Subsequently, elevated levels of OSTX indicate osteoblast maturation and the initiation of differentiation. Our results indicate that after 11 days of stimulation, the culture stimulated with 500 mV/mm shows a higher degree of maturation and differentiation, presenting higher levels of OSTX with respect to the rest of the conditions. The expression level of RUNX2 did not change significantly, which indicates that there were no changes in the early maturation stages. These results coincide with those of other authors [48, 49] and may suggest that these changes have occurred at an earlier stage of the stimulation and that at 11 days most of the cells are already differentiated, this hypothesis agrees with the high levels of OSTX found. Another possibility is that the positive activation of osteogenesis through electrical stimulation is regulated by other molecules such as BMP2 [26].

OPG is produced in response to different stimuli and is responsible for orchestrating bone remodelling in osteoblast cells [50, 51]. After the electrical stimulation, we did not observe changes in the mRNA levels, but we believe that it would be convenient in future studies to determine the RANKL values to verify the RANKL/OPG ratio, responsible for bone remodelling.

### Limitations, further investigation and applications

The results presented in this article show that direct current electrical stimulation can promote the differentiation of the studied cell lines (N2a and MC3T3), this differentiation can be optimized through the selection of the applied electric field amplitude. The effect of alternating current on this parameter has not been studied yet and it will be explored by the authors in the future, as a way to have a full picture of each cell line sensitivity to electric fields. Of course, a wide range of stimulation parameters could be explored, such as stimulation times, smaller/larger than 3 hours, higher amplitudes, different frequencies, and other waveforms. Other authors have shown that more than 15 days of stimulation can improve the MC3T3 results of proposed assays. Finally, a full biological characterization of N2a cells submitted to ES should be done, including a real time PCR analysis and other biochemical techniques.

The final objective of our project [SYMAS] is to control the ES conditions according to the evolution of the cell culture, so in the first phase of the project, the effects of different ES conditions are evaluated and classified. Once the ES conditions are tuned to ensure optimum control of the biological tissue, this technique could be used in vivo for nerve regeneration or bone growth in injuries or implants. Since neurites can be polarized, they can be directed to fill in injuries or repair damaged areas as some authors have done [16]. As we have previously said, ES could also be used to improve the osteointegration of implants, this is one of the most important challenges when implanting a prosthesis, especially with metal prosthesis that substitute bones; the authors of [18] have studied this application. All these applications are in a very early stage; we pretend to enhance even more the effects of the ES by optimizing its parameters to better affect the biological tissue over time.

## Conclusions

The present study shows that DC electrical stimulation (ES), in the range of 125-500 mV/mm, can promote differentiation and neurite outgrowth and reorganization in mouse neuroblastoma cells (N2a), it can also promote proliferation and differentiation of mouse pre-osteoblasts cells (MC3T3). The setup and protocols proposed in this study have produced the desired effects on the studied cell lines, as hypothesized based on literature. ES has been proven as a suitable tool for promoting differentiation of precursor cell cultures and could be used in tissue engineering and regenerative medicine procedures as a means of accelerating tissue regeneration and precursor cell specialization in bone or nervous system injuries.

## Data Availability

The datasets used and/or analyzed during the current study are available from the corresponding author on reasonable request.
